# Association between NOx exposure and deaths caused by respiratory diseases in a medium-sized Brazilian city

**DOI:** 10.1590/1414-431X20154396

**Published:** 2015-09-29

**Authors:** A. C. G. César, J. A. Carvalho, L. F. C. Nascimento

**Affiliations:** 1 Instituto Federal de Educação, Ciência e Tecnologia de São Paulo, Bragança Paulista, SP, Brasil; 2 Faculdade de Engenharia de Guaratinguetá, Universidade Estadual Paulista Júlio de Mesquita Filho, Guaratinguetá, SP, Brasil; 3 Departamento de Medicina, Universidade de Taubaté, Taubaté, SP, Brasil

**Keywords:** Air pollution, Nitrogen oxides, Respiratory diseases, Death

## Abstract

Exposure to nitrogen oxides (NOx) emitted by burning fossil fuels has been associated with respiratory diseases. We aimed to estimate the effects of NOx exposure on mortality owing to respiratory diseases in residents of Taubaté, São Paulo, Brazil, of all ages and both sexes. This time-series ecological study from August 1, 2011 to July 31, 2012 used information on deaths caused by respiratory diseases obtained from the Health Department of Taubaté. Estimated daily levels of pollutants (NOx, particulate matter, ozone, carbon monoxide) were obtained from the Centro de Previsão de Tempo e Estudos Climáticos Coupled Aerosol and Tracer Transport model to the Brazilian developments on the Regional Atmospheric Modeling System. These environmental variables were used to adjust the multipollutant model for apparent temperature. To estimate association between hospitalizations owing to asthma and air pollutants, generalized additive Poisson regression models were developed, with lags as much as 5 days. There were 385 deaths with a daily mean (±SD) of 1.05±1.03 (range: 0-5). Exposure to NOx was significantly associated with mortality owing to respiratory diseases: relative risk (RR)=1.035 (95% confidence interval [CI]: 1.008-1.063) for lag 2, RR=1.064 (95%CI: 1.017-1.112) lag 3, RR=1.055 (95%CI: 1.025-1.085) lag 4, and RR=1.042 (95%CI: 1.010-1.076) lag 5. A 3 µg/m^3^ reduction in NOx concentration resulted in a decrease of 10-18 percentage points in risk of death caused by respiratory diseases. Even at NOx concentrations below the acceptable standard, there is association with deaths caused by respiratory diseases.

## Introduction

Nitric oxide (NO), nitrous oxide (N_2_O) and nitrogen dioxide (NO_2_) generate nitrogen oxides (NOx), which are considered primary air pollutants, as well as carbon monoxide (CO), polycyclic aromatic hydrocarbons (PAHs) and particulates emitted from identifiable sources. These pollutants act as precursors of secondary air pollutants, such as ozone (O_3_), nitric acid (HNO_3_) and others (1). NOx also participate in the formation of photochemical smog consisting of products resulting from interaction with organic compounds and, together with sulfur dioxide (SO_2_), contribute to the formation of acid rain (2).

NOx formation can occur as the result of anthropogenic action, such as burning fossil fuels in stationary sources like industrial plants, or in motor vehicles (3). Most NOx is present as NO, but this species is readily oxidized to NO_2_ by reaction with O_3_, so NOx levels are similar to standard values for NO_2_ (4). NO_2_ is a very toxic gas that can trigger cell damage and inflammatory processes throughout the respiratory system, from the nose to the pulmonary alveoli (1).

It is estimated that diesel truck engines produce five times more NOx than lighter, gasoline-powered engines, per vehicle (5). Biodiesel use has contributed to the reduction in emissions of particulate matter (PM) and other pollutants (e.g., CO, PAHs); however NOx emissions have shown a slight increase even with the use of biodiesel blended with petroleum-based diesel (6). Studies project an annual growth rate of 3.5% in sales of smaller vehicles through 2015 and of 2.2% as from 2016, which would lead to increases of SO_2_, NO_2_ and PM emissions (7).

Mortality is among the effects of exposure to air pollutants. The risk of death associated with respiratory problems caused by exposure to all air pollutants is significantly higher than that of cardiovascular disease in 61% of cases, with 6-10% greater relative risk, on average (8). Data obtained by Goldberg et al. (9) indicated that individuals with certain health conditions, especially those with diabetes and cardiovascular disease, hypertension, atrial fibrillation and cancer, could be susceptible to the short-term effects of air pollution.

Saldiva et al. (10) examined the relationship between daily mortality in elderly adults (>65 years) and air pollution in the metropolitan area of São Paulo (SP), Brazil, for the period May 1990 to April 1991 by time series regression, controlling for season, weather, and other factors. Mortality was associated with respirable PM less than 10 µm in diameter (PM_10_), NOx, SO_2_, and CO. The association with PM_10_ was statistically significant, robust, and independent of other air pollutants. An increase in PM_10_ equal to 100 µg/m^3^was associated with an increase in overall mortality equal to approximately 13%.

Fine PM less than 2.5 µm in diameter [PM_2.5_], O_3_, and NO_2_ were found to have positive associations with mortality risk in a large cohort of California adults, using individualized exposure assessments (11). The positive associations with NO_2_ found in that study suggest that traffic pollution is related to premature death.

Decreases in air pollution may contribute to delaying deaths, as well as to reducing health costs and employee absenteeism; such decreases may also provide intangible benefits such as improved well-being, life expectancy and quality of life, as shown in a study with data from 25 European cities (12). Regression models have estimated the association between reductions in PM_2.5_ and improvements in life expectancy for the period 2000 to 2007 in 545 counties in the United States (13). Air pollution control over the last decade in that country has continued to have a positive impact on public health.

In studies conducted in São José dos Campos, a mid-sized city in the state of São Paulo, a decrease of 3 µg/m^3^ in the SO_2_concentration led to an 8.5% reduced risk of death from ischemic heart disease (14); also, exposure to air pollutants as a risk factor for death owing to stroke was identified, with increased risks of 10% and 7% for particulate matter and SO2, respectively (15).

The largest relative risk of death caused by respiratory disease in elderly adults was found by Martins et al. (16). Increments of 10 µg/m^3^ in daily levels of PM_10_ were associated with an overall 5.4% increase in respiratory mortality (relative risk [RR]=1.054, 95% confidence interval [CI]: 1.023-1.086), ranging from 1.4% (95%CI: 5.9-8.7) to 14.2% (95%CI: 0.4-28.0).

Increments of 10 µg/m^3^ in PM_10_ levels increased hospital admissions of elderly adults owing to respiratory illnesses by 3.5% (RR=1.035, 95%CI: 1.012-1.059) in Rio de Janeiro (17). In São Paulo (18), calculated RR=1.019 (95%CI: 1.011-1.027) and RR=1.009 (95%CI: 1.005-1.013), respectively for hospitalization and death in elderly adults was caused by respiratory diseases associated with a 10 µg/m^3^ increase in PM_10_.

The high industrialization rate of mid-sized cities in the Paulista Paraíba Valley of Brazil, and the associated increases in industrial vehicles and traffic on highways crossing that region prompted this study, aimed at estimating the association between exposure to NOx, represented by NO2, and mortality owing to respiratory diseases in residents of Taubaté, SP.

## Material and Methods

This was an ecological time-series study performed using records of deaths from respiratory diseases (ICD 10th revision: J12.0 to J18.9, J44.0 to J44.9, J45.0, J45.1, J45.8, J45.9 and J46) among residents of Taubaté, SP (of all ages and both sexes), between August 1, 2011 to July 31, 2012.

The geographical location of Taubaté is between São Paulo and Rio de Janeiro (23°02′S, 45°35′W), with a population of approximately 290,000. The city is crossed by the Presidente Dutra highway, the most important road in Brazil, with a high traffic flow of buses, cars and heavy trucks. Vehicle numbers in 2012 were estimated at about 180,000 cars and motorcycles and 7000 buses and trucks (19).

The Ethics Committee on Human Research of the Universidade de Taubaté (UNITAU) approved the study (#068/2012). Data on mortality owing to respiratory diseases were obtained from death certificates stored at the Epidemiological Surveillance Unit, which is linked with the Health Department of Taubaté.

Estimated daily levels of air pollutants were obtained from the Coupled Aerosol and Tracer Transport model to the Brazilian developments on the Regional Atmospheric Modeling System (CATT-BRAMS), considering daily means quantified as CO (ppb) and O_3_, NOx and PM_2.5_ (µg/m^3^). This system is an online transport model fully coupled to the CATT-BRAMS atmospheric model and has been designed to study the emission, deposition and transport of gases and aerosol particles using satellite data. The system generates daily estimates every 8 hours for different pollutants that are associated with biomass burning in South America (20). One of the advantages of using this model is its application to cities where there are no pollution measuring stations (20).

Data on temperature and humidity were obtained from the Centro de Previsão de Tempo e Estudos Climáticos website and the apparent temperature, which is a function of temperature and humidity, was calculated (21).

Models with exposure lags of 0 to 5 days (lag 0 to lag 5) were used to estimate the association between exposure to environmental pollutants and deaths associated with respiratory diseases. It is known that the effects of exposure to pollutants can be delayed, although there is no consensus about this interval. Therefore, we used the generalized additive Poisson regression model for analysis because deaths are discrete events.

The analysis consisted of modeling trends and seasonality in the series using spline functions for time; for weekdays and holidays, dummy variables were used. Inclusion and exclusion of terms in the model were evaluated for final fit and model quality. The corresponding terms for daily concentrations of pollutants were added to the model, assuming that the association with the dependent variable was linear.

The coefficients provided by the model can be translated into risk of death from respiratory diseases caused by exposure to pollutants, adjusted for apparent temperature. These relative risks (RR) with 95% confidence intervals, according to 0-5 day lags, were determined.

The estimated effects corresponding to a decrease of 3 µg/m^3^ in NOx levels were transformed into percentage decrease in risk of death caused by respiratory illness, with the respective 95% confidence intervals. A significance level of 5% was adopted for all analyses.

## Results

During the study period, there were 385 deaths owing to respiratory diseases, with 90% of these among elderly adults over 60 years old. The daily mean was 1.05±1.02, ranging from 0 to 5 deaths per day, regardless of age and sex.

The mean values with standard deviations, minimum and maximum values, and interquartile differences of atmospheric and pollutant variables are reported in Table 1. Mean values of pollutants PM_2.5_, NOx, O_3_ and CO did not exceed the standard set by Brazil’s State Decree No. 59113/2013, based on guidelines established by the World Health Organization (WHO) establishing new air quality standards through a gradual and progressive set of targets, such that air pollution is reduced to acceptable levels over time (22). Current standards for NO_2_ and PM_2.5_ are 60 µg/m^3^ and 20 µg/m^3^, respectively, considering the annual arithmetic mean. The standards for O_3_ and CO are 140 µg/m^3^ and 9 ppm, respectively, with sampling time of 8 h.

**Table:**
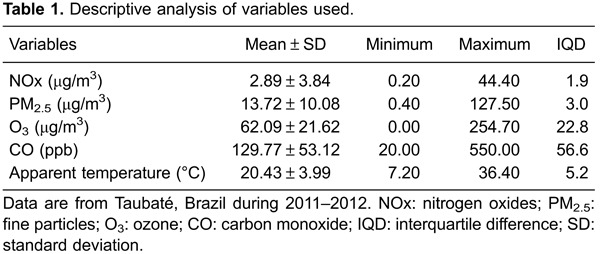

Poisson regression was used to obtain a model with all pollutants (multipollutant model) adjusted for apparent temperature, controlled by weekday and seasonality. This model provided the coefficients and SEs (Table 2) to calculate the RR of death from respiratory diseases after exposure to pollutants, and 95% confidence intervals, with lags of 0 to 5 days (Table 3).

**Table:**
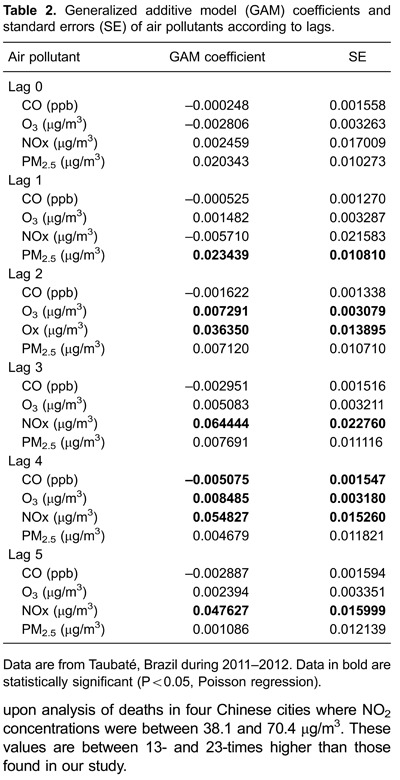

**Table:**
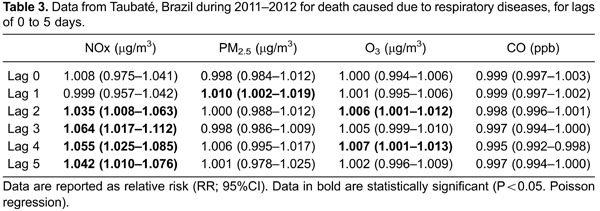

**Figure 1:**
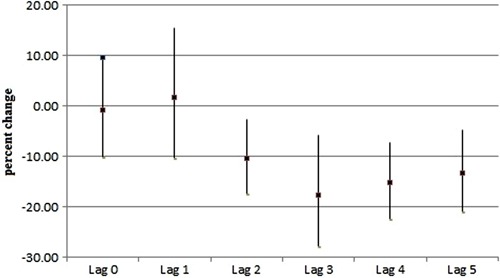
Percentage decrease of relative risks, and 95% confidence intervals of death owing to respiratory diseases with respect to nitrogen oxides (NOx) exposure and lag structure in Taubaté, Brazil (2011-2012).

Deaths were associated with exposure to PM_2.5_ in lags 0 and 1, to O_3_ in lags 2 and 4, and to NOx in lag 2 up to lag 5. A decrease of 3 µg/m^3^ in NOx concentrations implies a decreased risk of death between 11.5% (95%CI: 2.8-21.0) and 17.9% (95%CI: 7.8-28.9), corresponding to lags 2 and 3 (Figure 1).

## Discussion

An association between exposure to NOx and deaths from respiratory disease was observed in Taubaté, although the levels of NOx reported in this study were below those established by Cetesb (22).

Pollutant concentrations were not directly quantified by environmental agency measurements, but estimated by model; estimated data have been used in other studies (23-25). CO and PM_2.5_ near-surface measurements from CATT-BRAMS were validated (26). In studies such as Andrade Filho et al. (27), PM_2.5_levels were estimated by aerosol optical thickness measurements from the MODerate Resolution Imaging Spectroradiometer (MODIS) sensor.

NOx emitted in the form of NO are rapidly oxidized to generate NO_2_; thus, concentrations of NO_2_ can accurately reflect NOx concentrations (28). Among the results of exposure to NO_2_ are harmful effects on the respiratory tract, with impaired defenses against microorganisms and increased bronchial response in persons with asthma (28). Moreover, this pollutant is used as a marker of air pollution from burning fossil fuels. It is correlated with the presence of other pollutants generated by mobile sources and is the precursor of tropospheric O_3_ and nitrates that make up the fine fraction of particulate matter (PM_2.5_).

Studies have shown that gaseous air pollutants such as O_3_ and NO_2_ induce permeability of bronchial epithelial cells in primary cultures, with increased release of inflammatory mediators, thereby demonstrating that bronchial epithelial cells from patients with chronic airway diseases, such as asthma and chronic obstructive pulmonary disease, are more susceptible to the deleterious effects of air pollutants (29).

The results from our study are consistent with those of Tao et al. (30), which showed an increased risk of death up to 2 days after exposure to NO_2._ Increases of 10 µg/m^3^ in concentrations of this pollutant increased risk by about 3%, upon analysis of deaths in four Chinese cities where NO_2_ concentrations were between 38.1 and 70.4 µg/m^3^. These values are between 13- and 23-times higher than those found in our study.

In a nationally representative cohort in China, long-term exposure to outdoor air pollution was found to be associated with increased risk of cardiopulmonary and lung cancer deaths. Using proportional hazards regression models, an increase of 10 µg/m^3^ in NOx corresponded to 2.6% increased respiratory mortality (95%CI: −0.2% to 5.6%) (31), which is proportionally similar to that observed in our study. From 1991 to 2000, annual NOx concentrations in Chinese cities increased 28%, suggesting that air pollution gradually changed during the 1990s from the conventional coal combustion type to the mixed coal combustion/motor vehicle emission type (31), the same as what has occurred in Brazil.

In a study involving 17 Chinese cities with NO_2_ concentrations of 26 to 67 µg/m^3^, the risk of death caused by respiratory diseases increased by 2.52% (95%CI: 1.44-3.59), with higher risk in older people when concentrations of this pollutant increased 10 µg/m^3^ (32). Similar results were observed in our study, with 90% of deaths involving adults over 60 years of age.

A review of Chinese studies by Shang et al. (33) identified 11 reports on mortality associated with respiratory disease and NO_2_ exposure. The values found for risk of death were significant, ranging between 0.5% and 1.6%.

Wang et al. (34) used a time series method to construct an autoregressive integrated moving average (ARIMA) model of pollutants, and then inputted the predicted value of pollutants to a neural network model. This study was aimed at predicting the level of pollutants and concluded that the effect of NOx on the death rate owing to respiratory diseases is the greatest of all pollutants in Beijing.

Hospitalization for respiratory diseases in adults and children has also been described as a result of NO_2_ exposure (35,36). An increase of 10 µg/m^3^ in NO_2_ levels was associated with an increase in hospitalizations among elderly adults; RR=1.012 (95%CI: 1.007-1.017) for respiratory diseases, RR=1.024 (95%CI: 1.015-1.034) for chronic obstructive pulmonary disease, and RR=1.008 (95%CI: 1.002-1.014) for pneumonia (35).

The relationship between exposure to NO_2_ and hospitalization for pneumonia in children of Sorocaba, SP, Brazil, was statistically significant on the same day (RR=1.016; 95%CI: 1.007-1.025). This was also the case for particulate matter with a lag of 4 days (RR=1.009; 95%CI: 1.002-1.016) after exposure to pollutants (36).

In another study, researchers used data of children collected in different regions of Japan from 1997 to 2009. They observed that reductions in the concentrations of NO_2_ and suspended particulate matter, brought about by enforcement of measures to control automobile emissions, contributed to a decrease in the prevalence of respiratory diseases (37).

The actual levels of air pollution to which the population is exposed may be a limitation of this study. This is becausewe used modeled data instead of actual observations. Therefore, deaths may not be directly attributable to exposure to atmospheric pollutants (38). In contrast, this study reinforces the findings of other studies (14,36,39) that even populations in medium-sized cities can be affected by environmental pollution, which contributes to higher rates of morbidity and mortality.

This time series ecological study also has other limitations, such as a lack of individual information on exposure and disease. This study assumes homogeneous exposure throughout the city and that all individuals were similarly exposed. Another characteristic of this study is that we used records related to hospitalizations or deaths in the public health system. Thus, results reflect the effects of air pollutants on the portion of the population that uses the Unified Health System network, which is the majority of the Brazilian population (40).

The analysis performed in this study identified that exposure to NOx may be associated with deaths caused by respiratory diseases in a medium-sized city, even with pollutant emissions that are below the established standard. This information may serve as a warning to managers to expand investment in mass transport systems, which indirectly contribute to the improvement of public health.

## References

[1] Díaz Cónsul JMD, Thiele D, Veses RC, Baibich IM (2004). Decomposição catalítica de óxidos de
nitrogênio. Quim Nova.

[2] Teixeira EC, Feltes S, Santana ERR (2008). Estudo das emissões de fontes móveis na região
metropolitana de Porto Alegre, Rio Grande de Sul. Quim Nova.

[3] Cançado JED, Braga A, Pereira LAA, Arbex MA, Saldiva PHN, Santos UP (2006). Repercussões clínicas da exposição è poluição
atmosférica. J Bras Pneumol.

[4] Hernandez MF (2013). Estudio del impacto en la calidad del aire de las
fuentes pontual es en la ciudad de Pinar Del Río. Rev Bras Meteorol.

[5] Gaffney JS, Marley NA (2009). The impacts of combustion emissions on air quality and
climate - From coal to biofuels and beyond. Atmos Environ.

[6] Lin YC, Cheng MT, Ting WY, Yeh CR (2006). Characteristics of gaseous HNO_2_,
HNO_3_, NH_3_ and particulate ammonium nitrate in an urban
city of central Taiwan. Atmos Environ.

[7] Brasil (2015). Ministério do Meio Ambiente - MMA. 1° Inventário Nacional de
Emissões Atmosféricas por veículos automotores rodoviários. Diretoria de Mudanças
Climáticas, Secretaria de Mudanças Climáticas de Qualidade Ambiental, Ministério
do Meio Ambiente.

[8] Vanos JK, Hebbern C, Cakmak S (2014). Risk assessment for cardiovascular and respiratory
mortality due to air pollution and synoptic meteorology in 10 Canadian
cities. Environ Pollut.

[9] Goldberg MS, Burnett RT, Stieb DM, Brophy JM, Daskalopoulou SS, Valois MF (2013). Associations between ambient air pollution and daily
mortality among elderly persons in Montreal, Quebec. Sci Total Environ.

[10] Saldiva PH, Pope CA, Schwartz J, Dockery DW, Lichtenfels AJ, Salge JM (1995). Air pollution and mortality in elderly people: a
time-series study in Sao Paulo, Brazil. Arch Environ Health.

[11] Jerrett M, Burnett RT, Beckerman BS, Turner MC, Krewski D, Thurston G (2013). Spatial analysis of air pollution and mortality in
California. Am J Respir Crit Care Med.

[12] Pascal M, Corso M, Chanel O, Declercq C, Badaloni C, Cesaroni G (2013). Assessing the public health impacts of urban air
pollution in 25 European cities: results of the Aphekom project. Sci Total Environ.

[13] Correia AW, Pope CA, Dockery DW, Wang Y, Ezzati M, Dominici F (2013). Effect of air pollution control on life expectancy in
the United States: an analysis of 545 U.S. counties for the period from 2000 to
2007. Epidemiology.

[14] Amancio CT, Nascimento LF (2012). Association of sulfur dioxide exposure with circulatory
system deaths in a medium-sized city in Brazil. Braz J Med Biol Res.

[15] Amancio CT, Nascimento LF (2014). Environmental pollution and deaths due to stroke in a
city with low levels of air pollution: ecological time series
study. São Paulo Med J.

[16] Martins MC, Fatigati FL, Vespoli TC, Martins LC, Pereira LA, Martins MA (2004). Influence of socioeconomic conditions on air pollution
adverse health effects in elderly people: an analysis of six regions in São Paulo,
Brazil. J Epidemiol Community Health.

[17] Gouveia N, Mendonça GAS, Ponce de Leon A, Correia JEM, Junger WL, Freitas CU (2003). Poluição do ar e efeitos na saúde nas populações de duas
grandes metrópoles brasileiras. Epidemiol Serv Saúde.

[18] Gouveia N, Hajat S, Armstrong B (2003). Socioeconomic differentials in the temperature-mortality
relationship in Sao Paulo, Brazil. Int J Epidemiol.

[19] BGE (2014). Instituto Brasileiro de Geografia e
Estatística. Cidades.

[20] Freitas SR, Longo KM, Dias MAFS, Chatfield R, Dias PLS, Artaxo P (2007). The coupled aerosol and tracer transport model to the
Brazilian developments on the Regional Atmospheric Modeling System (CATT-BRAMS).
Part 1: Model description and evaluation. Atmos Chem Phys Discuss.

[21] Barnett AG, Tong S, Clements AC (2010). What measure of temperature is the best predictor of
mortality?. Environ Res.

[22] São Paulo (2014). Companhia de Tecnologia de Saneamento Ambiental
(CETESB). Qualidade do ar. http://www.bdlaw.com/assets/htmldocuments/Sao20Paulo20Decree2059113-2013.pdf.

[23] Ignotti E, Valente JG, Longo KM, Freitas SR, Hacon SS, Netto PA (2010). Impact on human health of particulate matter emitted
from burnings in the Brazilian Amazon region. Rev Saúde Pública.

[24] Cesar AC, Nascimento LF, Carvalho JA (2013). [Association between exposure to particulate matter and
hospital admissions for respiratory disease in children]. Rev Saúde Pública.

[25] Silva AM, Mattos IE, Ignotti E, Hacon SS (2013). Particulate matter originating from biomass burning and
respiratory. Rev Saúde Pública.

[26] Fuzzi S, Decesari S, Facchini MC, Cavalli F, Emblico L, Mircea M (2007). Overview of the inorganic and organic composition of
size-segregated aerosol in Rondônia, Brazil, from the biomass burning period to
the onset of the wet season. J Geophys Res.

[27] Andrade Filho VS, Artaxo P, Hacon S, Carmo CN, Cirino G (2013). Aerosols from biomass burning and respiratory diseases
in children, Manaus, Northern Brazil. Rev Saúde Pública.

[28] WHO (World Health Organization) (2005). Air Quality Guidelines for Particulate Matter, Ozone, Nitrogen
Dioxide and Sulfur Dioxide: Global Update 2005.

[29] Bayram H, Dikensoy O (2006). [Effects of air pollution on respiratory
health]. Tuberk Toraks.

[30] Tao Y, Huang W, Huang X, Zhong L, Lu SE, Li Y (2012). Estimated acute effects of ambient ozone and nitrogen
dioxide on mortality in the Pearl River Delta of southern China. Environ Health Perspect.

[31] Cao J, Yang C, Li J, Chen R, Chen B, Gu D (2011). Association between long-term exposure to outdoor air
pollution and mortality in China: a cohort study. J Hazard Mater.

[32] Chen R, Samoli E, Wong CM, Huang W, Wang Z, Chen B (2012). Associations between short-term exposure to nitrogen
dioxide and mortality in 17 Chinese cities: the China Air Pollution and Health
Effects Study (CAPES). Environ Int.

[33] Shang Y, Sun Z, Cao J, Wang X, Zhong L, Bi X (2013). Systematic review of Chinese studies of short-term
exposure to air pollution and daily mortality. Environ Int.

[34] Wang Q, Liu Y, Pan X (2008). Atmosphere pollutants and mortality rate of respiratory
diseases in Beijing. Sci Total Environ.

[35] Gouveia N, de Freitas CU, Martins LC, Marcilio IO (2006). [Respiratory and cardiovascular hospitalizations
associated with air pollution in the city of São Paulo, Brazil]. Cad Saúde Pública.

[36] Negrisoli J, Nascimento LF (2013). Atmospheric pollutants and hospital admissions due to
pneumonia in children. Rev Paul Pediatr.

[37] Hasunuma H, Ishimaru Y, Yoda Y, Shima M (2014). Decline of ambient air pollution levels due to measures
to control automobile emissions and effects on the prevalence of respiratory and
allergic disorders among children in Japan. Environ Res.

[38] Esquivel GAR, Gomes J, Grauer AF (2011). Evaluation of the correlation between atmospheric
pollutant concentrations and elderly mortality in Curitiba. Eng Sanit Ambient.

[39] Oliveira MS, Leon AP, Mattos IE, Koifman S (2011). Differential susceptibility according to gender in the
association between air pollution and mortality from respiratory
diseases. Cad Saúde Pública.

[40] Gavinier S, Nascimento LFC (2014). Air pollutants and hospital admissions due to
stroke. Rev Ambient Água.

